# Safety and Tolerability of Accelerated Venom Immunotherapy with Depot Extracts in Insect-Venom-Allergic Patients

**DOI:** 10.3390/medicina62081579

**Published:** 2026-08-17

**Authors:** Olga Branicka, Ewelina Krupa-Lubas, Andrzej Bożek, Tomáš Balner, Radosław Gawlik

**Affiliations:** 1 Clinical Department of Internal Diseases, Allergology and Clinical Immunology in Katowice, Medical University of Silesia, 40-752 Katowice, Poland; 2 Clinical Department of Internal Disease, Dermatology and Allergology in Zabrze, Medical University of Silesia, 41-800 Katowice, Poland; 3 Department of Allergology and Clinical Immunology, University Hospital Ostrava, University of Ostrava, 708-52 Ostrava, Czech Republic; 4 Institute of Laboratory Medicine, Faculty of Medicine, University of Ostrava, 703-00 Ostrava, Czech Republic

**Keywords:** venom immunotherapy, rush immunotherapy, depot allergen extract, hymenoptera venom allergy, safety

## Abstract

*Background and Objectives*: Hymenoptera venom allergy (HVA) represents one of the most frequent causes of anaphylaxis in adults. Venom immunotherapy (VIT) remains the only causal intervention that effectively reduces the risk of systemic reactions following subsequent stings. Accelerated-induction protocols enable rapid attainment of the maintenance dose; however, data on their safety with depot preparations are still limited. To evaluate the safety of accelerated-induction VIT using a depot preparation in a real-world clinical setting. *Materials and Methods*: A retrospective, single-center study was conducted between 2022 and 2024; accelerated-induction VIT with a depot preparation was initiated in 54 consecutive patients at a tertiary referral center. All patients had a documented history of systemic allergic reactions after Hymenoptera stings. A three-day accelerated-induction protocol was applied. Safety outcomes and adverse events occurring during the induction phase were systematically recorded and analyzed. *Results*: All 54 patients successfully achieved the maintenance dose. Adverse reactions were observed only in five patients (9.3%) and were restricted to mild local reactions at the injection site, including erythema and localized swelling. No systemic reactions or cases of anaphylaxis were reported. None of the patients required permanent discontinuation of VIT. *Conclusions*: Accelerated-induction VIT using a depot preparation demonstrated a favorable safety profile. When performed in an experienced tertiary care setting, this approach allows for rapid attainment of the maintenance dose while maintaining excellent tolerability.

## 1. Introduction

Hymenoptera venom allergy (HVA) represents one of the most common causes of anaphylaxis in the adult population [1,2]. Hypersensitivity to hymenoptera venom most commonly presents with local reactions, including large local reactions; however, it may also lead to systemic reactions that constitute a potentially life-threatening condition [3,4]. Epidemiological evidence indicates that deaths related to Hymenoptera stings in Europe are rare but well documented. Analysis of data from the WHO Mortality Database identified 1691 sting-related deaths coded as ICD-10 X23 (contact with hornets, wasps and bees) across 32 European countries between 1994 and 2016 [5]. According to the World Allergy Organization (WAO), despite the observed increasing trend in hospitalizations, mortality due to anaphylaxis among patients allergic to Hymenoptera venom is estimated at 0.09–0.13 deaths per million inhabitants per year. However, it should be remembered that even the first anaphylactic reaction to Hymenoptera venom may result in death, which usually occurs within 30–60 min, regardless of the amount of venom injected [1,3]. Systemic allergic reactions represent the most clinically relevant manifestation of Hymenoptera venom allergy and can occur after a single sting [4,6].

Venom immunotherapy (VIT) has been used in clinical practice for many decades. The first reported case of desensitization in a patient allergic to Hymenoptera venom was described in 1925 [7], while the first randomized controlled trial demonstrating the efficacy of VIT was published in 1978 [8,9]. Since then, numerous studies have confirmed the high efficacy of venom immunotherapy. VIT remains the only disease-modifying therapeutic intervention that significantly reduces the risk of systemic reactions following subsequent stings, with reported efficacy of 91–96% for wasp venom and 77–84% for honeybee venom [10,11].

Venom immunotherapy is associated with adverse reactions, which occur most frequently during the induction phase [11]. In an EAACI multicenter study, adverse reactions were reported in approximately 20% of patients, although most were mild and self-limiting [12]. Systematic reviews indicate a higher risk of adverse reactions during honeybee venom immunotherapy compared with wasp venom immunotherapy [13]. Traditionally, the induction phase of venom immunotherapy was performed using aqueous venom extracts. In the search for safer ways to achieve a maintenance dose during VIT, attention turned to depot preparations [11,13] used earlier only in maintenance phase of VIT. When the availability of aqueous extract (Pharmalgen) had decreased and its production was finally definitively stopped, we decided to use the available depot solution—Alutard SQ—during the initial phase of VIT. Following positive experience with cluster regimens in aeroallergen immunotherapy, where the initial build-up phase with increasing doses of three different strengths was shortened by reducing the conventional escalation protocol to a regimen using only the highest concentration [14], we decided to implement this in venom immunotherapy.

The induction phase of venom immunotherapy may be performed using ultra-rush, rush, cluster, or conventional protocols. Accelerated schedules allow for a shorter period of incomplete protection and reduce the number of visits during the initial phase of treatment, the reason for many concerns raised by patients. Venom preparations are available as aqueous solutions and as aluminum-hydroxide-adsorbed depot preparations [13,15]. Previous studies have demonstrated comparable safety profiles of aqueous and depot preparations, with some evidence suggesting potential advantages of depot extracts, particularly in honeybee venom allergy [10,13]. Although depot preparations have traditionally been used mainly in conventional protocols, emerging evidence supports their use in accelerated-induction schedules when administered in experienced centers [16,17,18].

## 2. Materials and Methods

This retrospective, single-center study was conducted at the Department of Internal Medicine, Allergology and Clinical Immunology, University Clinical Hospital K. Gibiński in Katowice, Poland. Medical records of patients qualified for venom immunotherapy (VIT) between 2022 and 2024 were analyzed. Qualification for VIT was based on a documented history of systemic allergic reaction following a Hymenoptera sting (severity 3 i 4 acc EAACI), together with evidence of sensitization to the corresponding venom, demonstrated by the presence of venom-specific IgE and/or positive skin prick and intradermal test results. Skin test (ST) was performed for *Apis mellifera*, *Vespula* spp.: skin prick tests were carried out with undiluted aqueous solution (100 mg/mL, Venomen, HAL, Leiden, The Netherlands) and intradermal tests with 0.02 mL of diluted solution. Maximum non-irritant venom concentrations were 1–190 mg/mL [6].

Exclusion criteria included the absence of confirmed Hymenoptera venom allergy, uncontrolled asthma, pregnancy at the initiation phase of VIT, previous venom immunotherapy, and incomplete clinical or laboratory documentation required for confirming HVA. Patients with mastocytosis were not excluded; however, baseline serum tryptase levels were assessed in all patients as part of the risk evaluation. All patients had serum tryptase levels within the normal reference range, and no elevated tryptase values suggestive of an underlying mast cell disorder were identified. Patients qualified for VIT were informed of desensitization protocols and asked to choose a traditional, cluster or accelerated procedure. To more quickly achieve protection and to save time, most patients preferred the accelerated schedule.

In the present study, patients underwent a three-day up-dosing protocol during hospitalization. Purified depot preparations of either Alutard SQ bee venom (ALK-Abelló, Hørsholm, Denmark) or Alutard SQ wasp venom (ALK-Abelló, Hørsholm, Denmark) were used. Concentrations 1–4 contained 100, 1000, 10,000 and 100,000 SQ-U/mL, respectively. On the first day, Alutard was administered from concentration 1 at a dose of 0.1 mL up to concentration 2 at a dose of 0.3 mL. On the second day, Alutard concentration 3 was administered at doses ranging from 0.1 mL to 0.3 mL, and on the third day, concentration 4 was administered at doses ranging from 0.1 mL to 0.3 mL. The cumulative dose administered during the three-day inpatient phase was 64,440 SQ-U, corresponding to approximately 64.4 μg of the appropriate insect venom. Injections were administered at 30–60 min intervals. Following the final daily injection, patients remained under continuous clinical observation throughout the remainder of their hospitalization, with 24 h inpatient monitoring. Premedication with antihistamines or systemic corticosteroids was not routinely used. All patients achieved the maintenance dose during hospitalization. Further patient management, including subsequent dose escalation, was conducted in an outpatient setting. The subsequent visit took place 7 days after the previous one, during which a dose of 0.6 mL was administered. The following visit occurred after a 14-day interval, at which time a dose of 0.8 mL of concentration 4 was administered. Thereafter, dose escalation was continued according to individual clinical tolerance until the prescribed maintenance dose (1.0 mL) was reached. After each dose administered in the outpatient setting, patients were observed for up to 60 min for the occurrence of any immediate adverse reactions. All patients ultimately reached their prescribed maintenance dose. They received a dose that should protect them in the event of a sting by a live insect, preventing life-threatening anaphylaxis from occurring.

The Bioethics Committee of the Medical University of Silesia in Katowice issued a statement confirming that this study did not require approval from the Bioethics Committee (No. BNW/NWN/0052/KB/236126).

## 3. Results

Fifty-four patients (23 women) underwent VIT using the proposed accelerated protocol. Of these, 18 patients received honeybee venom and 36 received wasp venom. The clinical characteristics of the study population are summarized in Table 1, whereas the severity of previous anaphylactic reactions is presented in Table 2. Comorbid conditions were common in the study population; cardiovascular diseases were more frequent among patients receiving wasp venom immunotherapy (21 patients) compared with those receiving honeybee venom (6 patients). Diabetes mellitus and chronic respiratory diseases were less prevalent (five and four patients, respectively). No clear association between the use of ACEIs or beta-blockers and the occurrence of adverse reactions during honeybee or wasp venom immunotherapy was observed in the analyzed cohort.

All 54 patients completed the up-dosing phase and continued maintenance treatment without the need for dose modification or treatment discontinuation. According with the current EAACI guidelines for venom immunotherapy [11], mild reactions during the induction phase were observed in five patients (9.3%), exclusively among those treated with wasp venom, and these were limited to local reactions at the injection site, including erythema and local swelling requiring only antihistamines and local treatment (Table 3). No generalized reactions or cases of anaphylaxis were observed.

## 4. Discussion

In a traditional schedule of allergen immunotherapy, weekly injections of increasing doses of allergen extract are required for about 12–16 weeks (initial build-up phase) to reach a maximum maintenance dose of allergen. To overcome this inconvenience, accelerated schedules of allergen immunotherapy (AIT) have been developed whereby the initial build-up phase is completed within 1–3 days. The use of accelerated immunotherapy has been associated with a faster onset of clinical benefits and significant acceptance of the patients but also with a risk of side effects [19,20,21].

Similar strategies of accelerated up-dosing with depot allergen preparations have previously been explored in aeroallergen immunotherapy. A randomised pediatric study compared standard dose escalation with Novo-Helisen^®^ Depot through strengths 1–3 with an abbreviated schedule initiated directly with strength 3. The abbreviated approach reduced the number of injections and the time required for induction, while the overall adverse event profile, including systemic reactions, remained similar between the study groups. No serious treatment-emergent adverse events were recorded, and successful attainment of the maintenance dose among participants completing escalation further supported the practical feasibility of this approach. Because the study involved a different allergen and clinical indication, its results cannot establish the safety of accelerated VIT; nevertheless, they show that simplified induction may be achievable with another aluminum-hydroxide-adsorbed allergen preparation [14].

Earlier European recommendations did not support the use of depot extracts in rapid schedules, partly because conventional regimens were considered better tolerated and rapid dose escalation had been associated with an increased risk of adverse reactions [22]. The subsequent EAACI guidelines similarly noted that aluminum-hydroxide-adsorbed preparations were typically administered using conventional or cluster schedules and emphasised that their apparently favorable local tolerability might partly reflect the slower build-up schedules employed rather than an inherent safety advantage of the formulation itself [11]. Nevertheless, accelerated VIT was not considered inherently unsafe, as modified rush protocols had been reported to represent acceptable alternatives to standard weekly schedules when patients were appropriately selected and supervised [23]. Thus, the traditional association of depot preparations with prolonged up-dosing appears to reflect historical practice and limited experience with accelerated depot-based protocols rather than definitive evidence that these formulations require slower administration [11,22,23].

However, recent studies have demonstrated that depot preparations may also be used in accelerated-induction protocols with acceptable safety and tolerability when administered under close supervision in experienced allergy units [16,17,24]. These observations challenge the traditional restriction of depot extracts to prolonged conventional schedules. Nevertheless, the available protocols differ considerably in duration, dosing intensity and treatment setting, and the current evidence does not identify a single preferred accelerated approach. These studies therefore provide an important clinical context for interpreting the results of the more condensed, three-day inpatient protocol evaluated in the present study.

In this study, accelerated inpatient build-up of venom immunotherapy using an aluminum-hydroxide-adsorbed depot preparation demonstrated a favorable tolerability profile. All 54 patients successfully completed the three-day inpatient build-up phase, subsequently continued dose escalation in an outpatient setting and ultimately reached their prescribed maintenance dose. Adverse reactions occurred in five patients (9.3%) and were limited exclusively to local injection-site reactions. Importantly, no systemic reactions or cases of anaphylaxis were observed during the induction phase.

Alternative three-day rush protocols using aluminum-hydroxide-adsorbed depot venom preparations have also been described, including a protocol in which concentrations 1–3 were administered on the first day and concentration 4 was introduced on the second day, resulting in a cumulative inpatient venom dose of 161.1 μg. By comparison, our protocol involved a more gradual progression, with concentrations 1 and 2 administered on the first day, concentration 3 on the second day and concentration 4 introduced on the third day. Based on the administered volumes and the stated concentrations of the preparations, the cumulative dose during our three-day inpatient phase was 64,440 SQ-U, corresponding to approximately 64.4 μg of venom. Stoevesandt and Trautmann reported large local reactions in three patients (5.1%) and one moderate systemic reaction during honeybee VIT following administration of a 20 μg dose. In our cohort, adverse reactions occurred in five patients (9.3%) and were limited exclusively to local reactions, with no systemic reactions observed. Direct comparison of the local reaction rates is limited by differences in their definitions: the previous study included only reactions exceeding 10 cm in diameter and persisting for at least 24 h, whereas our analysis included local injection-site reactions more broadly. Despite differences in dose escalation and cumulative venom exposure, both studies support the feasibility of a three-day inpatient build-up using aluminum-hydroxide-adsorbed depot preparations under appropriate clinical supervision. However, their observational designs, relatively small cohorts and lack of direct comparator groups preclude definitive conclusions regarding comparative safety [25].

The rate of adverse reactions observed by our group during the up-dosing phase (9.3%) is comparable to or even lower than rates reported in previous observational studies of venom immunotherapy, where adverse events during induction have been described in approximately 20% of patients, with the majority being mild and self-limiting [10,11,12]. According to EAACI guidelines and large cohort studies, systemic reactions occur predominantly during the buildup phase and are more frequently reported in patients treated with honeybee venom compared with wasp venom [10,11,26]. Notably, in the present study, no systemic reactions were observed despite the inclusion of patients undergoing honeybee venom immunotherapy, which supports the favorable tolerability of depot preparations in accelerated protocols.

Accelerated- and cluster-induction schedules are increasingly used to shorten the period of incomplete protection and reduce the number of visits required during the initial phase of venom immunotherapy [11,23]. However, experience with accelerated protocols using aluminum-hydroxide-adsorbed extracts has traditionally been limited, as these preparations have historically been used mainly in conventional up-dosing regimens [13,22,27]. Cadavid-Moreno et al. reported that all patients achieved the maintenance dose by day 7 with a favorable tolerability profile, with only mild and transient local or systemic reactions observed and no delayed reactions reported [16]. Kasternow et al. demonstrated the safety of an accelerated immunotherapy protocol using an aluminum-hydroxide-adsorbed Hymenoptera venom depot in a 7-week protocol, achieving the maintenance dose [18].

Brunello et al. started VIT with the highest concentration of depot venom extract of 100,000 SQ and observed only one case of mild systemic reaction. All treated patients tolerated the initial phase of VIT well [24].

Shortening the inpatient build-up phase may reduce the number of treatment visits, facilitate earlier progression towards maintenance treatment and decrease the organizational burden associated with VIT for both patients and healthcare providers. These practical advantages could potentially improve treatment acceptance, adherence and patient-reported quality of life.

An important strength of this study is its real-world design, reflecting routine clinical practice rather than a highly selected trial population. Patients with relevant comorbidities, including cardiovascular diseases and arterial hypertension, were included, which aligns with everyday clinical decision-making in tertiary allergy centers. Moreover, premedication with antihistamines or systemic corticosteroids was not routinely applied, indicating that the favorable safety profile observed was not dependent on pharmacological pre-treatment.

Several limitations should be acknowledged. The single-center setting may also limit the generalizability of the findings. Furthermore, the absence of a comparator group made it impossible to determine whether the observed outcomes differed from those obtained with aqueous venom preparations or with conventional build-up using the same aluminum-hydroxide-adsorbed depot preparation. The relatively small sample size may have been insufficient to detect very rare adverse events. Finally, protection against subsequent field stings was not systematically evaluated, although the effectiveness of VIT in preventing systemic sting reactions is well established [10,11]. Therefore, although the present findings support the feasibility and favorable tolerability of the investigated protocol, its comparative safety and clinical effectiveness require confirmation in larger prospective controlled studies. Accelerated protocols reduce the number of hospital admissions, resulting in lower costs for both the patient and the health system.

## 5. Conclusions

Accelerated-induction venom immunotherapy with a depot preparation demonstrated a favorable tolerability profile in real-life clinical practice. In this cohort of 54 patients, all individuals achieved the maintenance dose, and adverse events were limited to mild local reactions. The absence of generalized reactions and anaphylaxis supports the feasibility of this approach when performed by experienced allergists in appropriately equipped medical settings. However, its comparative safety requires validation and confirmation in larger prospective controlled studies.

Protection was not investigated in this part of the study and is an aim of further observation, as is the absence of any reactions after field stings by the culprit insect in all resting patients.

## Figures and Tables

**Table 1:** Characteristics of patients qualified for venom immunotherapy.

	All	Bee Venom	Wasp Venom
*n* (%)	54	18 (33)	36 (67)
Age (years; mean ± SD)	51.3 ± 14.4	48.0 ± 17.3	52.9 ± 12.6
Male *n* (%)	31 (57)	11 (61)	20 (56)
Female *n* (%)	23 (43)	7 (39)	16 (44)
Co-morbidity *n* (%)			
Cardiovascular disease	27 (50)	6 (33)	21 (58)
Respiratory disease	4 (7)	1 (6)	3 (8)
Diabetes mellitus	5 (9)	1 (6)	4 (11)
Cancer	2 (4)	0	2 (6)
Cardiovascular drugs *n* (%)			
Beta-blockers	11 (20)	2 (18)	9 (82)
ACEIs	8 (14)	4 (50)	4 (50)
Occupational exposure	5 (9)	5 (28)	0

**Table 2:** Baseline severity of anaphylactic reactions according to Ring and Messmer.

Severity of Anaphylactic Reactions	All	Bee Venom	Wasp Venom
I Skin and subcutaneous manifestations (generalized erythema, urticaria, angioedema)	8	3	5
II Multisystem involvement (skin symptoms and hypotension, tachycardia, dyspnea, or gastrointestinal symptoms)	10	4	6
III Life-threatening symptoms (severe anaphylaxis: shock, severe bronchospasm, cardiovascular collapse)	10	5	5
IV Cardiac and/or respiratory arrest	26	6	20

**Table 3:** Symptoms during up-dosing phase.

Symptoms Up-Dosing Phase	All	Bee Venom	Wasp Venom
No side effects	49	18	31
Local reactions (large) (%)	5 (9.3)	0	5 (9.3)
Objective systemic symptoms	0	0	0

## Data Availability

The raw data supporting the conclusions of this article will be made available by the authors on request.
